# Participation as a Pillar of Active Ageing: The Role of Eudaimonic Psychological and Health Factors

**DOI:** 10.3390/geriatrics10010011

**Published:** 2025-01-13

**Authors:** Teresa Paniagua-Granados, Virginia Fernández-Fernández, María Ángeles Molina-Martínez

**Affiliations:** 1 Department of Psychology, Universidad Europea de Madrid, 28670 Madrid, Spain; 2 Department of Psychology of the Personality, Evaluation and Psychological Treatments, Universidad Nacional de Educación a Distancia (UNED), 28040 Madrid, Spain; mmolina@psi.uned.es

**Keywords:** active ageing, older adults, participation, eudaimonic well-being, health

## Abstract

**Background/Objectives:** With ageing population projections, promoting positive ageing trajectories is critical. While health is often emphasised, eudaimonic psychological factors remain underexamined. A qualitative study presented throughout the main text highlighted the importance of psychological factors like purpose in life and resilience in fostering participation and subjective well-being, even amidst declining health. This model bridges the most recent updates from governmental organisations—the International Longevity Center, Brazil and the World Health Organization. Building on this model, the current research seeks to empirically assess the impact of health and eudaimonic psychological factors on the frequency and satisfaction of participation among older adults. **Methods:** This study involved 289 participants (56.74% women) aged 65+ in Madrid. Data on participation, self-perceived health, and eudaimonic factors were collected through an online survey. Hierarchical regression and cluster analyses explored the predictors and profiles of participation. **Results:** Resilience, positive relationships, and autonomy explained 8.8% of variance in participation frequency. Satisfaction was influenced by health, meaning in life, and autonomy, accounting for 11% of variance. Profiles showed the highest participation and satisfaction in individuals with high eudaimonic scores, despite moderate health. **Conclusions:** Eudaimonic factors significantly influence participation and mitigate health limitations, reinforcing the qualitative study model mentioned. By uniting updates from governmental organisations proposals, this model underscores the role of psychological well-being in active ageing. Future research should explore hedonic well-being as a key outcome of active ageing.

## 1. Introduction

Demographic studies show an increase in the number and proportion of older people worldwide, a trend that is projected to keep rising [1]. Spain is one of the European countries with the highest life expectancy, where older people represent 20% of the population (INE, 2022) and will continue increasing to 27.4% in 2040 [2] and 36.8% in 2050 [3].

Against this backdrop, promoting positive ageing trajectories is considered a major challenge [4]. This approach is based on the fact that (1) the forms of ageing vary significantly and can be divided into three main types— normal, pathological and active, or successful [5,6]; (2) these types of ageing do not occur randomly, and there are individual and contextual determinants that influence ageing trajectories [7].

The World Health Organisation defined active ageing (AA) as “the process of optimising opportunities for health, participation and security to improve the quality of life of ageing persons” [7]; subsequently, it added a new pillar, lifelong learning, and culture, gender, behavioural factors, environment, social and economic aspects, health and social services, and personal factors were identified as determinants [8].

According to the literature, several attempts have been made to operationalise the AA model empirically, using both a quantitative [9,10,11,12] and qualitative approaches [13,14], without reaching a consensus about its formulation [15]. Disparities exist in the types and numbers of variables and measures and instruments considered in the different models [10,11,12], and some dimensions are emphasised over others, both in the pillars—such as health [16] or, especially, participation [17,18,19]—and in the determinants [20,21] or in the outcome variables to which they have been related [22,23].

This scenario poses problems that include recognising the importance of the different components and determinants of the AA process. In this regard, less attention has been paid to psychological variables [24].

**Figure 1:**
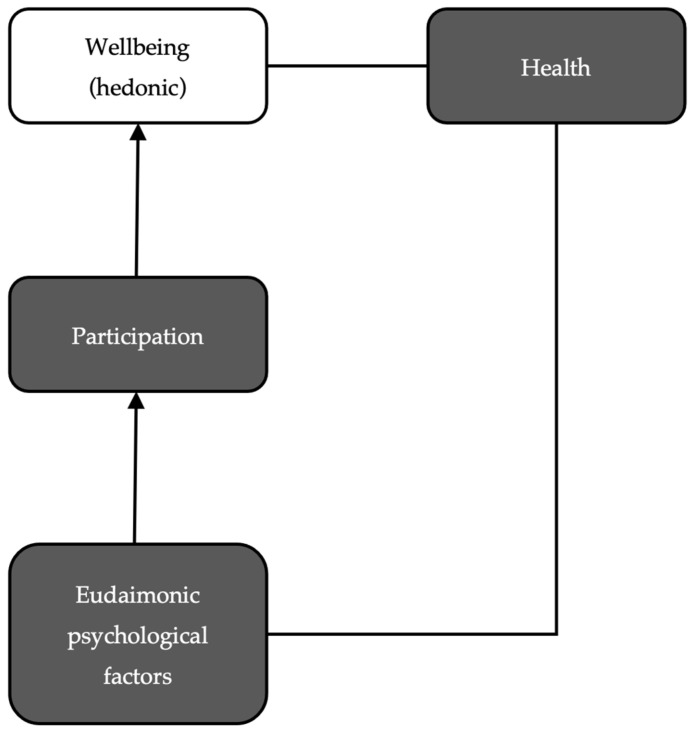
Model obtained with qualitative analysis [25].

In an attempt to further understand the AA model, a qualitative study was conducted in which this conception was analysed from a non-academic perspective [25]. To obtain further knowledge about the role of psychological variables in the AA model, a content analysis was carried out to replicate the WHO model and discover the weight of psychological factors in the lay conception [25]. The analysis found that psychological variables were recognised in the discourses analysed both as outcome variables and as determinants and mediators of the process. From that analysis, it was found that the outcome of active ageing is “feeling good”, the means to achieve it is participation—learning activities are assumed to be a type of participation—and the determinant of participation is eudaimonic psychological variables, such as purpose in life or adaptation. As regards the pillars of the model, older people understand that health is likely to worsen in the course of ageing, but it will not condition participation when it interacts with eudaimonic variables. These conclusions are summarised in Figure 1.

These conclusions are relevant, not only because of the recognition of psychological factors, but also because they identify (personal) factors with which to intervene to promote positive ageing trajectories. Moreover, the resulting model overcomes salutogenic reductionism and recognises the value of participation in old age as a pathway to (hedonic) well-being. These conclusions are similar to the healthy ageing (HA) model proposed by the WHO [26]: the outcome is also well-being and, and to achieve this, it is considered essential to promote functional ability, which means the “attributes that enable people to be and to do what they have reason to value” [26] (p. 28), which is similar to the concept of participation.

Given the importance of participation, it is necessary to delve into its definition and the factors associated with it, as there is a great deal of controversy in the literature in this regard. Thus, it is necessary to verify whether the results obtained in the aforementioned qualitative study [25] regarding the variables that predict participation are empirically confirmed. This would allow us to identify the variables that require intervention to promote participation in older people and, consequently, achieve positive ageing trajectories.

Participation means “engaging in any social, civic, recreational, cultural, intellectual or spiritual pursuits that provide meaning, fulfilment and a sense of belonging” [8] (p. 47). Although most research has focused on assessing whether or not older people participate [27], many others recognise the importance of the subjective feeling that participation provides for each individual [28,29]. A distinction must be made between an objective and a subjective dimension of participation [30]. The objective component can be operationalised as the frequency of participation and is the most studied [27]. Frequency of participation is crucial, given the negative impact on health of sedentary lifestyles, particularly in older people [31]. The subjective dimension, understood as satisfaction with participation, has been researched less [29,31]. People’s satisfaction with their participation is associated with higher life satisfaction, a powerful indicator of quality of life [28]. Consequently, studying both dimensions contributes to gaining a better understanding of participation [31], leading to more complete knowledge of the factors that promote AA.

As regards the variables that foster participation, the non-academic proposal of Molina-Martínez et al. [25] defines components such as purpose in life or adaptation. These variables are encompassed within the eudaimonic conception of well-being, understood as “the process and achievement of those values that make us feel alive and authentic, that make us grow as people and not so much in the activities that give us pleasure or keep us away from pain” [32] (p. 154). The WHO’s reformulation of the AA model [8] mentions these types of factors among the personal determinants of well-being, citing two theoretical models. The first is Ryff’s model of psychological well-being [33,34], which distinguishes six variables to be considered: autonomy, personal growth, self-acceptance, purpose in life, mastery of the environment and ability to relate positively. The second is the PERMA model [35] which, in its conceptualisation of well-being, considers both hedonic and eudaimonic variables such as commitment, meaning in life and achievement. Psychological eudaimonic variables are mentioned not only in the two most recent proposals published by governmental agencies [8,26] but also in other qualitative studies on positive ageing trajectories [36]. Moreover, they have been linked to older people’s participation, with evidence in the literature that they are factors that promote their engagement in activities they consider to be of value [37,38,39,40].

In this sense, the study by Molina-Martínez et al. [25] concludes that the presence of these psychological factors will interact with health, such that deteriorating health, as a consequence of the ageing process, will not have a detrimental impact on participation. The relationship between participation and health has usually been considered from a two-way perspective: just as being active in old age leads to better health outcomes, being healthy leads older people to engage in more activities [41,42,43]. However, it is important to take into consideration that being in good health is not a necessary condition for engaging in activities and occupying leisure time in a satisfying and fulfilling way for older people. In this sense, participating in activities is a mediator between older people’s physical health and their physical and emotional well-being [44]. Only some health problems negatively impact certain types of activities. In addition, numerous theories, such as the SOC Model [45], argue that older people, faced with physical deterioration, manage to deploy several strategies that let them overcome deficits and engage very positively. The real challenge, therefore, is for older people to be motivated to fill their leisure time by participating in activities that are rewarding and meaningful to them.

This study therefore aims to explore the role of physical health and eudaimonic psychological variables in (1) the frequency of participation and (2) satisfaction with participation in a sample of Spanish older people. Thus, it is hypothesised that both physical health and eudaimonic variables will have a combined and significant effect on the frequency and satisfaction of participation, with psychological variables acting as mediators in this relationship.

## 2. Materials and Methods

### 2.1. Participants

The sample was selected in the Madrid Region (Spain) from a universe of people aged 65 and over. The inclusion criteria were being over 64 years of age, being able to read and write and being able to respond autonomously to a computer interview. The characteristics of the sample (*n* = 289) were as follows: 125 men with a mean age of 73.63 years (range: 65–90); 4.9% reported having no regular education (although they could read and write), 14.4% had a primary education, 35.3% a secondary education, 15.2% higher education and 30.2% university education. Concerning marital status, 59.2% reported being married, 11.4% were single, 10.6% divorced and 17.1% widowed. A total of 74.3% of the sample considered that their economic conditions were sufficient to cope with day-to-day life.

### 2.2. Procedure

The information was accessed using the C.A.W.I. (computer-assisted online interview) system. All participants were informed of the objectives of the study and consented to participate. The study was approved by the Universidad Francisco de Vitoria Ethics Committee (registration number: 34/2019) on 6 November 2019.

### 2.3. Variables and Instruments

#### 2.3.1. Dependent Variable: Participation

Frequency of participation. Frequency of participation in eight community and individual activities (e.g., volunteering; attending training or educational courses; attending a sports, social or other club; reading books, magazines or newspapers; playing word or number games such as crosswords or Sudoku) was surveyed with a Likert-type response format (1 = almost daily, 2 = every week, 3 = every month, 4 = less frequently and 5 = never) [46]. After inverting the items, the variable “frequency of participation” was calculated as the sum of the frequency of each of the activities.

Satisfaction with participation. Information was collected about older people’s satisfaction with participation in these activities using the question “On a scale of 0 to 10 where 0 means totally dissatisfied and 10 means totally satisfied, how satisfied are you with the activities you mentioned?”

#### 2.3.2. Independent Variables: Eudaimonic Psychological Factors and Health

##### Health

Perceived health was assessed with the question that assesses self-perceived health taken from the adaptation of the PERMA Scale [47] by Paniagua-Granados et al. [48]: “In general, how would you say your health is”. The response format is Likert-type with 11 points (0 = very bad to 10 = excellent). Higher scores on this question indicate better perceived health.

##### Eudaimonic Psychological Factors

Purpose in life, mastery of the environment, positive relationships, self-acceptance, autonomy and personal growth were measured using the six subscales of the Spanish adaptation [49,50] of Carol Ryff’s psychological well-being scale [33]. The Spanish adaptation includes 29 items, and each subscale consists of 4 to 6 items, with a Likert-type response format (from 0 = strongly disagree to 5 = strongly agree). High scores suggest high levels of psychological well-being in each of the variables mentioned. Good reliability coefficients (Cronbach’s Alpha) were obtained in this study: αself-acceptance = 0.84; αautonomy = 0.72; αgrowth = 0.71; αmastery = 0.71; αpurpose = 0.85; αrelationships = 0.78. Similar results were found in the Spanish validation, with Alpha values ranging from 0.70 (autonomy and purpose in life) to 0.84 (self-acceptance) [49,50].

Meaning in life, achievement and commitment were assessed using the corresponding subscales of the Spanish older population adaptation of the PERMA Scale [47] conducted by Paniagua-Granados et al. [48]. Each of them contains 3 items (e.g., “In general, to what extent do you lead a purposeful and meaningful life?”, “How long do you feel you are making progress in your life?” and “In general, to what extent do you feel excited and interested in things?”). The response format is on an 11-point scale with anchor labels: (1.) from 0 (never) to 10 (always); (2.) from 0 (not at all) to 10 (completely). High scores on each subscale suggest the presence of higher levels of the variables assessed. In this study, good to acceptable Cronbach’s alpha coefficients were obtained, with values of 0.85 (meaning in life), 0.68 (achievement) and 0.61 (commitment). The coefficients found in the original version were 0.90 (meaning in life), 0.79 (achievement) and 0.71 (commitment) [47].

Resilience, understood as willingness to cope with stress in a highly adaptive manner, was measured using the Brief Resilient Coping Scale (BRCS; [51]). It is a 4-item questionnaire with a 5-choice Likert-type response format (1 = does not describe me at all; 5 = describes me very well), validated in Spanish older adults [52]. The internal consistency found in the Spanish validation with older adults was high, similar to this study, with a Cronbach’s Alpha of 0.84.

### 2.4. Data Analysis

The inferential analyses were preceded by an atypical case analysis [53] of the responses to the scales considered, as well as a response internal consistency analysis (Cronbach’s Alpha). In addition, the regression analysis assumptions proposed by Berry and Feldman [54] were tested, and a descriptive analysis (means, standard deviations, frequencies and percentages) of the socio-demographic variables and the variables of interest was carried out.

With the aim of exploring the role that health and eudaimonic variables play in participation, two strategies were followed: (1) conducting explanatory models through multiple regressions, and (2) investigating whether the sample could be classified into profiles based on their levels of eudaimonic and health variables, to then analyse if there were differences in participation between these profiles.

Thus, for inferential statistics purposes, two regressions were performed using the stepwise method, in which the model only includes entered variables that contribute significantly to explaining the dependent variable variance. The criterion variables in both models were frequency of participation and satisfaction with participation. In addition to using the stepwise method, the predictor variables were entered in both models in two steps, following the results obtained in the study by Molina-Martínez et al. [25]. Therefore, the health variable (perceived health) was entered in the first step, and the eudaimonic psychological factors, i.e., purpose in life, mastery of the environment, positive relationships, self-acceptance, autonomy, personal growth, sense of life, achievement, commitment and resilience, in the second.

Cluster analyses were performed to explore the sample grouping profiles in terms of health and eudaimonic psychological variables, starting with a first hierarchical cluster analysis to visually inspect the dendrogram, followed by a K-means cluster analysis, pre-setting the number of clusters to three based on the visual inspection already carried out. The results obtained for each variable in each cluster (health and eudaimonic variables) were classified into low, medium or high scores, depending on the scores obtained in the terciles carried out before. Mean difference analyses (one-way ANOVA) were performed for the outcome variables (frequency of participation and satisfaction with participation), according to each cluster. To ascertain between which clusters the statistically significant differences occurred, post hoc analyses were carried out, specifically Tukey’s HSD statistic, since Levene’s test indicated equality of variances in the variables. Finally, effect size measures were calculated, specifically eta squared.

All statistical analyses were carried out using IBM SPSS Statistics 23.

## 3. Results

### 3.1. Descriptive Analysis of Study Variables

Table 1 shows the descriptive statistics for the variables of interest.

### 3.2. Stepwise Multiple Regression Analysis

Table 2 shows the results of the hierarchical regression analysis to explain the frequency of participation of older people. The final model obtained is significant (F285 = 10.14; *p* = 0.001) and explains 8.8% of the total variance in the frequency of participation.

Table 3 shows the results of the hierarchical regression analysis of satisfaction with participation. The final model obtained is significant (F265 = 11.93; *p* = 0.001) and explains 11% of the total variance in satisfaction with participation. It should be noted that the variable perceived health ceases to significantly explain the variance in satisfaction when meaning in life is introduced, suggesting a possible mediating effect.

### 3.3. Cluster Analysis for Profile Identification

Table 4 shows the descriptive statistics of the three profiles identified after observation of the dendrogram, according to the perceived health of the participants and their scores for the eudaimonic psychological variables.

Table 5 shows the mean difference analyses (one-factor ANOVA) on the scores of the outcome variables (frequency of participation and satisfaction with participation) and as a function of the profiles (clusters) and suggests statistically significant differences in all the proposed variables. The effect size measures (eta squared) suggest an index of 0.02 for the frequency of participation variable and 0.04 for satisfaction with participation, with both effects being small.

**Figure 2:**
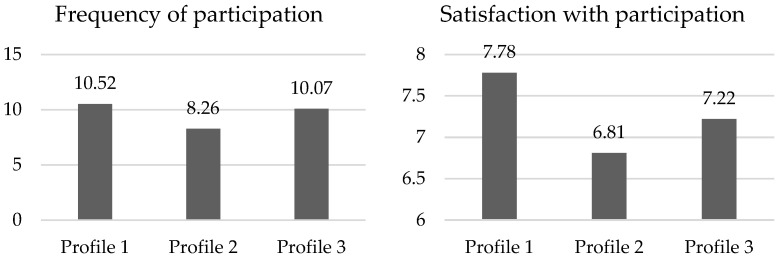
Mean scores for each group for outcome variables.

Figure 2 shows the mean scores of the groups for each of the outcome variables (frequency of participation and satisfaction with participation).

The post hoc analyses are shown in Table 6. This analysis of the frequency of participation differences indicates that they occur between groups 1 and 2, with higher scores for profile 1. As for the post hoc results for satisfaction with participation, the statistically significant differences are between groups 1 and 2 and between groups 1 and 3, with profile 1 showing higher levels of satisfaction in both cases.

## 4. Discussion

The main objective of this study was to explore the role of health and eudaimonic psychological variables in promoting participation, a pillar of AA. The point of departure was the need to empirically contrast some of the conclusions reached in the non-academic proposal of the AA model by Molina-Martínez et al. [25]. The results allow us to partially corroborate this research.

Given the lack of consensus in the literature when it comes to defining the participation pillar, this study addressed both its objective dimension (understood as frequency of participation) and subjective dimension (satisfaction with participation). The data support the richness and contribution to knowledge of considering both [31], as they appear to be explained by different variables.

The frequency of participation result show that eudaimonic variables predict part of its variance, namely resilience, positive relationships and autonomy. These findings re-emphasise what has already been highlighted in the latest updates on ageing models proposed by governmental agencies [8,26]. As previously addressed, the ILC-BR explicitly emphasises eudaimonic variables and, particularly, resilience [8]. Resilience is proposed as an evolutionary construct whose promotion or erosion can shed light on the AA process [8]. Regarding the WHO model [26], resilience is again related to what is defined under this approach as functional ability (the “attributes that enable people to be and to do what they have reason to value”) (p. 28). In addition, this variable is widely supported by scientific studies. Older people’s ability to adapt to adversity is associated with positive variables, such as engagement in meaningful activities [55]. The same is true for positive relationships with others [37,56] and autonomy [38,40], both of which have been linked to the frequency of older people’s participation. However, in the results obtained in the present study, perceived health is not part of the model, with the introduction of eudaimonic variables. Although the literature has usually shown that being in good health during ageing leads to increased activity engagement [41,42], this result is consistent with the proposal of Molina-Martínez et al. [25], as well as with the contributions of gerontological models such as the aforementioned SOC model [45]. In the resulting model, it seems that older people’s perception of being able to make decisions (i.e., their autonomy) weighs more than their health conditions.

These conclusions are also reached when comparing the frequency of participation of people in the three profiles found according to their levels of eudaimonic psychological factors and perceived health. The group with significantly higher participation frequency scores is the group of older adults with higher levels of eudaimonic variables, despite rating their perceived health with average scores. In contrast, the group with the lowest significant levels of participation in activities is the one in which people show low levels of all the variables considered. Once again, the modulating role of eudaimonic well-being variables in the frequency of participation is confirmed.

In the case of satisfaction with participation, the perceived health of older adults does enter the model, which is consistent with previously mentioned studies that have related health conditions to participation [41,42]. However, when introducing meaning in life, perceived health loses its significant value. This suggests that meaning in life might have a mediating effect on the relationship between health and participation. This idea is consistent with what older people said in the non-academic proposal by Molina-Martínez et al. [25], who stated that, although health would decline with age, their participation would not be conditioned if they encountered eudaimonic factors. Meaning in life has also been found to mediate the relationship between health and other mental health variables in ageing [57]. Therefore, in the presence of eudaimonic variables and, in particular, meaning in life, deteriorating health does not seem to impair the subjective dimension of participation. Thus, the eudaimonic variables that are part of this model are meaning in life and, again, autonomy, an effect found in other studies [38,39,40]. These results once again align with the role of psychological variables in the governmental publications described [8,26]. In the case of the ILC-BR model [8], the focus is placed on eudaimonic variables, as previously mentioned. Regarding the WHO model [8], meaning in life would fall under the umbrella of the description of functional capacity.

Once again, these conclusions are obtained in the comparisons of satisfaction with participation between the profiles obtained. The first profile, made up of those older people with higher levels in most of the eudaimonic variables and average levels of perceived health, shows significantly higher satisfaction with their participation than the other two, made up of adults with lower scores in the variables assessed.

While eudaimonic variables appear to play an important role in explaining older adults’ participation in both dimensions analysed, both the percentage of variance explained and the effect size measures are small. These findings show how complex it is to understand older people’s participation, and to further define the factors that promote it. A multitude of variables explain their engagement in activities, including personal interests [58], socio-cultural stereotypes related to ageing [59] or lack of opportunities to participate in activities [60].

Furthermore, most of the studies reviewed attempt to further understand the variables that promote social participation, whereas if one took the ILC-BR [8] definition of participation, it would be somewhat reductionist to focus only on participation in the community. Other types of individual activities should also be considered, as this study has aimed to do. However, future research may need to investigate further whether these two types of activities (individual and community) are explained by different variables and have a different impacts on psychoemotional well-being during ageing.

The limitations of this study include the sample itself, which is not representative due to its size and characteristics. The sample consisted of non-institutionalised older adults with a high level of education and familiarity with new technologies, which relates to the online format of the survey. It has been shown that people who tend to participate in this type of survey usually have more knowledge, affinity with the topic and commitment [61]. A bias in the representativeness of the sample is therefore identified, reducing external validity [62]. Clearly, not everyone has access to the Internet [63], and there may be important differences between the Internet access of middle- and low-income individuals. Therefore, the results presented in this paper should be taken with caution, as they introduce an important bias regarding the external validity of the work. Furthermore, the cross-sectional nature of the study suggests caution in considering the results achieved, and the development of longitudinal studies would be desirable to test the hypotheses.

One aim of future research is to empirically contrast the rest of the model [25] and ascertain whether participation results in the presence of hedonic well-being during ageing. This could be used to integrate the proposals of governmental bodies [8,26], providing an AA model in which psychological variables play a relevant role.

## 5. Conclusions

To conclude, eudaimonic variables seem to play a significant role in older people’s commitment to activities that they consider to be of value. This fact can be used to approach the empirical validation of the model of Molina-Martínez et al. [25] and to further identify those psychological factors that promote good ageing.

## Figures and Tables

**Table 1:** Descriptive analysis of study variables.

	Participation		P.H.	Eudaimonic Well-Being									
	F.P.	S.P.		P.G.	M.E.	L.P.	AT.	S-A.	P.R.	A	M.L.	C	R
N	289	268	289	288	289	288	289	289	289	289	289	289	289
M	9.99	7.48	7.04	14.66	18.20	15.11	20.84	14.94	17.40	7.20	7.27	7.25	15.07
SD	5.01	1.76	1.75	3.23	3.94	3.28	4.91	3.18	4.59	1.34	1.59	1.52	3.09
Range	0–40	0–10	0–10	0–20	0–25	0–25	0–30	0–20	0–25	0–10	0–10	0–10	0–20

F.P.—frequency of participation; S.P.—satisfaction with participation; P.H.—perceived health; P.G.—personal growth; M.E.—mastery of the environment; L.P.—life purpose; AT.—autonomy; S-A.—self-acceptance; P.R.—positive relationships; A—Achievement; M.L.—meaning in life; C—commitment; R—resilience.

**Table 2:** Stepwise multiple regression analysis for frequency of participation.

	Model	ΔR^2^	Beta	T	Simple Correlation	Semi-Partial Correlation	Collinearity Statistics	
							Tolerance	VIF
1	(Constant)	0.04 **		3.28 **				
	Resilience		0.20	3.54 **	0.20	0.20	1.00	1.00
2	(Constant)	0.02 *		1.89 **				
	Resilience		0.16	2.80 **	0.16	0.16	0.93	1.07
	Positive relationships		0.14	2.36 *	0.13	0.13	0.93	1.07
3	(Constant)	0.03 **		2.87 **				
	Resilience		0.22	3.62 **	0.21	0.20	0.87	1.15
	Positive relationships		0.22	3.51 **	0.20	0.19	0.80	1.26
	Autonomy		−0.22	−3.37 **	−0.19	−0.19	0.80	1.32

ΔR^2^: increase in percentage of variance explained; **: *p* ≤ 0.01; *: *p* ≤ 0.05.

**Table 3:** Stepwise multiple regression analysis for satisfaction with participation. ΔR^2^: increase in percentage of variance explained; **: *p* ≤ 0.01; *: *p* ≤ 0.05.

	Model	ΔR^2^	Beta	T	Simple Correlation	Semi-Partial Correlation	Collinearity Statistics	
							Tolerance	VIF
1	(Constant)	0.02 **		13.54 **				
	Perceived health		0.16	2.69 **	0.16	0.16	1.00	1.00
2	(Constant)	0.07 **		8.10 **				
	Perceived health		0.02	0.42	0.02	0.02	0.80	1.24
	Meaning in life		0.30	4.70 **	0.27	0.27	0.80	1.24
3	(Constant)	0.02 *		6.20 **				
	Perceived health		0.02	0.43	0.02	0.02	0.80	1.24
	Meaning in life		0.26	3.88 **	0.23	0.22	0.74	1.35
	Autonomy		0.14	2.31 *	0.14	0.13	0.90	1.10

**Table 4:** Descriptive statistics of profiles identified.

		Clusters					
		Profile 1 N = 152		Profile 2 N = 42		Profile 3 N = 92	
		Mean (SD)	Level	Mean (SD)	Level	Mean (SD)	Level
Perceived health		7.40 (1.68)	Medium	5.50 (1.85)	Under	7.24 (1.37)	Medium
Eudaimonic psychological factors	P.G.	16.34 (2.40)	High	10.83 (2.52)	Under	13.88 (2.38)	Medium
	M.E.	20.69 (2.57)	High	12.80 (2.63)	Under	16.88 (2.49)	Under
	L.P.	16.76 (2.26)	High	10.11 (2.80)	Under	14.86 (2.04)	Medium
	AT.	23.95 (3.30)	High	18.11 (4.63)	Under	16.97 (3.75)	Under
	S-A.	16.58 (2.04)	High	10.00 (2.65)	Under	14.65 (2.11)	Medium
	P.R.	20.33 (3.38)	High	13.92 (3.50)	Under	14.28 (3.25)	Under
	A	7.73 (1.09)	High	5.51 (1.25)	Under	7.15 (1.05)	High
	M.L.	7.94 (1.15)	Medium	5.12 (1.15)	Under	7.32 (1.21)	Medium
	C	7.69 (1.29)	Medium	5.70 (1.50)	Under	7.28 (1.38)	Medium
	R	16.27 (2.61)	High	11.71 (2.55)	Under	14.85 (2.58)	Medium

P.G.—personal growth; M.E.—mastery of the environment; L.P.—life purpose; AT.—autonomy; S-A.—self-acceptance; P.R.—positive relationships; A—achievement; M.L.—meaning in life; C—commitment; R—resilience.

**Table 5:** Mean differences in outcome variables according to clusters.

	Cluster	N	Mean	SD	Standard Error	*F*	*p*
Frequency of participation	Profile 1	152	10.52	4.58	0.37	3.42	
	Profile 2	42	8.26	4.76	0.73		0.03
	Profile 3	92	10.07	5.61	0.58		
Satisfaction with participation	Profile 1	152	7.78	1.77	0.14	5.97	0.00
	Profile 2	42	6.81	1.75	0.28		
	Profile 3	92	7.22	1.63	0.17		

**Table 6:** Differences between profiles in frequency of participation and satisfaction with participation.

Grouping of Clusters		Frequency of Participation			Satisfaction with Participation		
Profile	Profile	HSD	*p*	S.E.	HSD	*p*	S.E.
1	2	2.26	0.02	0.86	−0.97	0.00	0.31
	3	0.45	0.77	0.65	0.55	0.04	0.23
2	1	−2.26	0.02	0.86	0.97	0.00	0.31
	3	−1.81	0.12	0.92	−0.41	0.43	0.33
3	1	−0.45	0.77	0.65	−0.55	0.04	0.23
	2	1.81	0.12	0.92	0.41	0.43	0.33

HSD: Tukey’s HSD; S.E.: standard error.

## Data Availability

The data presented in this study are only available on request from the corresponding author due to ethical reasons.
